# The safety and efficacy of airway pressure release ventilation in acute respiratory distress syndrome patients: A PRISMA-compliant systematic review and meta-analysis

**DOI:** 10.1097/MD.0000000000018586

**Published:** 2020-01-03

**Authors:** Xuri Sun, Yuqi Liu, Neng Li, Deyuan You, Yanping Zhao

**Affiliations:** a Department of Critical Care Medicine, The Second Affiliated Hospital, Fujian Medical University, Quanzhou; b Department of Pathogenic Biology, School of Basic Medical Sciences, Fujian Medical University, Fuzhou, Fujian Province; c Department of Critical Care Medicine, Chinese Medicine Hospital Changji Autonomous Prefecture, Changji, Xinjiang Uygur Autonomous Region, PR China.

**Keywords:** acute respiratory distress syndrome, airway pressure release ventilation, mechanical ventilation

## Abstract

**Background:**

The acute respiratory distress syndrome (ARDS) is a critical illness with high mortality and a worse prognosis. Mechanical ventilation (MV) is currently considered to be one of the most effective methods of treating ARDS. In this meta-analysis, we discussed the efficacy of airway pressure release ventilation (APRV) in treating ARDS.

**Methods:**

Following the Preferred Reporting Items for Systematic Review and Meta-Analysis (PRISMA), Ovid Medline, Embase, and PubMed were systematically searched with the keywords of “ARDS” and “APRV”. The studies containing the treatment of APRV in ARDS were included. According to the MV protocol used in the studies, the comparison was undertaken between the APRV group vs low tidal volume (LTV) group and synchronized intermittent mandatory ventilation (SIMV) group. The relative risk (RR) and the standard mean difference with 95% confidence intervals (CI) were used for the comparison between groups.

**Results:**

Fourteen studies with 2096 patients were included in the meta-analysis. The average increasing rate of PaO_2_/FiO_2_ was 75.4% in the APRV group vs 44.1% in the non-APRV group. No significant differences were found in mortality and duration of ICU stay between APRV vs LTV (*P* = .073 and *P* = .404) and APRV vs SIMV (*P* = .370 and *P* = .894).

**Conclusion:**

The APRV protocol would have a higher increase in the PaO_2_/FiO_2_ ratio, which was a safe protocol with a compatible effect comparing to LTV and SIMV.

## 1 Introduction

Acute respiratory distress syndrome (ARDS) is a common critical illness that might lead to the multiple organ dysfunction syndrome (MODS) and even death.^[1]^ ARDS was first proposed by Ashbaugh in 1967, and it was characterized as refractory hypoxemia and severe respiratory distress.^[2]^ The clinicopathological aspects, which includes severe inflammatory injury to the alveolar-capillary barrier, surfactant depletion, and loss of aeratable lung tissue, immediately leads to profound hypoxemia, decreased lung compliance, and increased intrapulmonary shunt and dead space.^[3]^ Currently, it is suggested that ARDS is characterized by acute diffuse pulmonary inflammation and increased alveolar permeability during trauma, stress, and injury.^[4]^ However, the pathogenesis of ARDS is not fully understood, resulting in relatively limited treatment, which ultimately leads to a high mortality rate as high as 40% to 50%.^[5–7]^

In the clinical development of ARDS disease, refractory hypoxemia is the primary physiological feature. Therefore, providing active and adequate oxygen therapy has a positive effect on the prognosis of patients with ARDS. Invasive mechanical ventilation (MV) is currently considered to be one of the most effective methods of ARDS treatment.^[8]^ In the early stage of development of this technique, the standard MV mode was adopted, which utilize a normal tidal volume and conventional positive end-expiratory pressure (PEEP). However, for patients with ARDS, it was found that the suboptimal PEEP levels in MV can cause ventilator-induced lung injury, which is associated with higher mortality, extended ICU stay and high cost.^[9,10]^ Thereafter, the low tidal volume ventilation is widely used and considered as the current standard MV strategy.^[5]^

Airway pressure release ventilation (APRV) is a pressure controlled, intermittent mandatory ventilation mode with a short intermittent release phase, allowing the release of partial lung volume and spontaneous breathing throughout the high level of pressure.^[9,11]^ It has a purported advantage than conventional MV in alveolar recruitment, preservation of spontaneous breathing, improving oxygenation and hemodynamics, and potential lung-protection.^[11]^ Although it has been developed for almost 20 years, APRV is still not used as a routine for patients in clinical practice in several countries. Up to now, APRV is mainly used as rescue therapy for the patients with ARDS who cannot be properly oxygenated by MV.^[11]^ More recently, several studies demonstrated that the early application of APRV in ARDS patients could reduce the duration of ventilation and intensive care unit (ICU) stay with a improving oxygenation outcome.^[9,12]^ However, the efficacy of APRV in the patients diagnosed with ARDS is still controversial. Thus, we designed this systematical review and meta-analysis.

## 2 Methods

The PRISMA guidelines were used for designing this study,^[13]^ and this study was in accordance with the ethical guidelines of the *Declaration of Helsinki*.

### 2.1 Search strategy and study selection

The Ovid Medline, Ovid Embase, Cochrane Central Register of Controlled Trials, Cochrane Database of Systematic Reviews, and PubMed were systematically searched. The Google Scholar and related websites were also searched for grey literatures. The searching keywords were “airway pressure release ventilation”, “Bi-Vent”, “APRV” and “respiratory distress syndrome”. All the database was searched from its inception to 23rd January 2019. All the studies containing abstracts and titles were imported into Endnote (Clarivate Analytic, version X5) for duplicate papers exclusion and literature screening.

### 2.2 Inclusion and exclusion criteria

All the studies mentioned the use of APRV in ARDS in the database we searched were involved in our study. The inclusion criteria were:

(1) the study mentioned the approach of APRV;
(2) the dataset of patients containing the disease of APRV (complete or partial);
(3) the data of intensive care unit ventilation data or outcome could be extracted in the study;
(4) study design was limited in observational study, case-control study, cohort studies, and randomized control trials.

The meta-analysis, review, and comments were reading for further inclusion of studies. Only articles written in English were involved in this systematic review.

The exclusion criteria included

(1) animal experiment;
(2) the studies not mentioning ARDS or not mentioning the percentage of ARDS patients;
(3) case reports, conference abstract, and non-English studies;
(4) no available data of outcome or respiratory assessment;
(5) containing pediatric ARDS patients.

### 2.3 Literature Screening, data extraction, and quality assessment

Two investigators (XS and LY) independently screened the titles and abstracts according to the inclusion and exclusion criteria. If the inclusion and exclusion of the literature could be determined based on the criteria, the full text was further evaluated. The third reviewer (LN) was adapted for discussion if there were any disagreement existed between the former two investigators.

The following information was collected based on original studies. The study characteristics (author, publish year, recruitment period, study title, study design, institution, etc.), the ventilation mode (APRV and following mentioned mode), the characteristics of patients in different ventilation group (sample size, disease, age, gender, acute physiology and chronic health evaluation II score, cause of ARDS and etc.), the ventilator setting and respiratory measurement in baseline and Day 3 (ventilator parameters, airway pressure, Pa_O2_/FI_O2_, and etc.) and outcome assessment (mortality, tracheostomy, duration of ventilation, duration of ICU stay, duration of hospital stay and etc.) were extracted and collected.

Two investigators (YD and ZY) independently assess the quality of the included papers. For those randomized controlled trials (RCT), the Cochrane Collaboration tool for assessing the risk of bias in RCTs was used to evaluate the quality of the literature.^[14]^ Moreover, for those case-control and cohort studies, the Newcastle-Ottawa Scale (NOS) was used for assessing the quality.^[15]^

### 2.4 Definition of the disease and ventilation protocol

Until recently, the most common definition for ARDS was proposed by American-European Consensus Conference (AECC) in 1994, in which ARDS was defined as the acute onset of respiratory failure, bilateral infiltrates on chest radiograph, hypoxemia with the PaO_2_/FiO_2_ ratio ≤ 200 mmHg without the evidence of arterial hypertension or cardiogenic edema.^[16]^ Currently, the Berlin definition was accepted globally in 2013, which defined the ARDS as an acute diffuse, inflammatory lung injury, with PaO_2_/FiO_2_ ratio ≤300 mmHg while ratio >200 mmHg was categories as mild ARDS.^[4]^ Thus, in our study, studies with acute lung injury (ALI) patients were included.

The different ventilation modes and their definitions were summarized in Table 1.^[17,18]^ The primary modes, such as volume-control ventilation (VCV), pressure-control ventilation (PCV), and pressure support ventilation, were used to compare the efficacy with APRV. Moreover, the combination ventilation mode including assist-control ventilation (A/C), synchronized intermittent mandatory ventilation (SIMV), continuous positive airway pressure, and low tidal volume (LTV) ventilation were also compared. Generally speaking, for both LTV ventilation and APRV, the mechanical ventilation goals were to achieve adequate oxygenation with required PaO_2_ and can be weaned to less than 55 mmHg as tolerated, and arterial pH > 7.25.

**Table 1:**
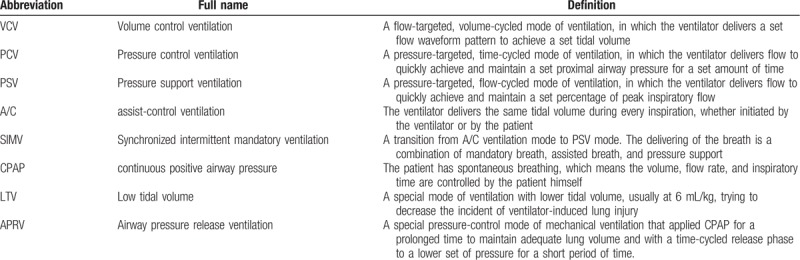
The ventilation protocol mentioned in the meta-analysis.

### 2.5 Statistically analysis

The relevant outcomes of this meta-analysis were performed using Stata (version 15.0 Stata Corporation, College Station, TX). The relative risk (RR) was used for statistical analysis for categorical variables, while for continuous variables, the standard mean difference (SMD) was used. Both were reported with 95% confidence intervals (CI) and the P value was set as 0.05. If there were data provided as medians and range (or interquartile range), we would convert the data into means and standard deviation (SD) using the formula provided by Hozo et al.^[19]^ The heterogeneity was evaluated using the I^2^ statistic and χ^2^ test was used for statistical heterogeneity (I^2^ ≥ 50% indicating the presence of heterogeneity). When the heterogeneity existed, the random-effects model was used, while on the contrary, the fix-effect model was used. Finally, the forest plots were drawn, and the funnel plots were used for evaluating the publication bias.

## 3 Results

### 3.1 Literature selection

**Figure 1:**
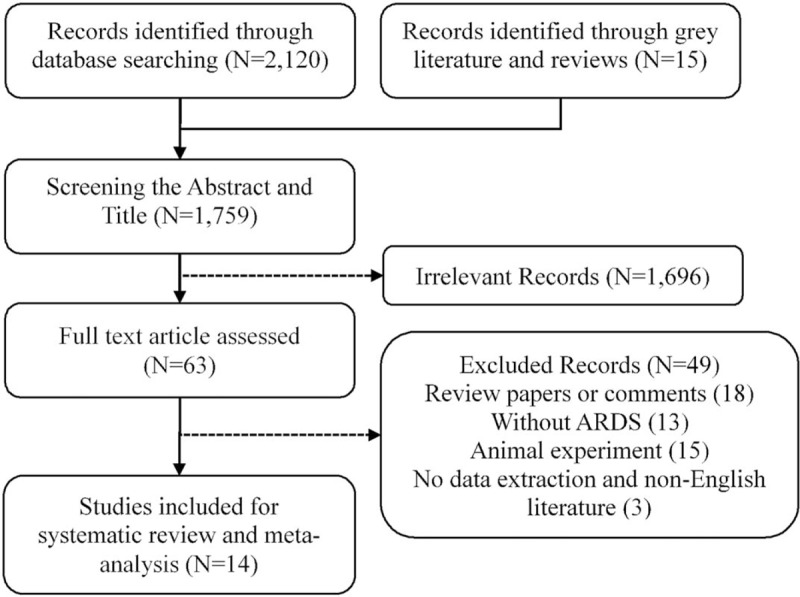
Flowchart of literature screening of the meta-analysis.

A total of 2135 studies were found by search strategy. The flowchart was shown in Figure 1. After screening the abstracts and titles, 63 studies were screened in full text. After excluding the animal experiment, studies mentioned no ARDS, case reports and non-English literature, 14 studies with 2,096 patients were finally included in the meta-analysis.^[5–7,9,12,20–28]^

### 3.2 Characteristics of the selected studies

The characteristics of the included studies were summarized in Table 2. Among them, 7 studies were RCTs and 3 studies were retrospective case-control studies. The rest 4 studies were observational studies or crossover studies without a control group. This systematic review includes 13 single centers study from 6 countries (China, Japan, USA, Australia, Germany, and Finland), and one multi-center studies from 23 countries. Three studies compared the APRV vs LTV protocol.^[5,9,12]^ Four studies compared APRV vs SIMV protocol.^[7,21,25,26]^ The rest 3 studies compared ARPV vs A/C, PSV, and PCV, respectively.^[6,22,27]^ The median age of APRV group patients were 51.5 years old (range 40–70 years old), while non-APRV group patients were 52 years old (range 42–63 years old). The median acute physiology and chronic health evaluation II (APACH II) score were 21 vs 20 in APRV and non-APRV group. The baseline median PaO_2_/FiO_2_ was 113 (range 78–215) in APRV group vs 131 (range 96 to 220) in the non-APRV group. And on Day 3, the median PaO_2_/FiO_2_ was 190 (range 150–280) in APRV group vs 180 (range 161–212) in the non-APRV group. The average increasing rate of PaO_2_/FiO_2_ was 75.4% in APRV group vs 44.1% in the non-APRV group.

**Table 2:**
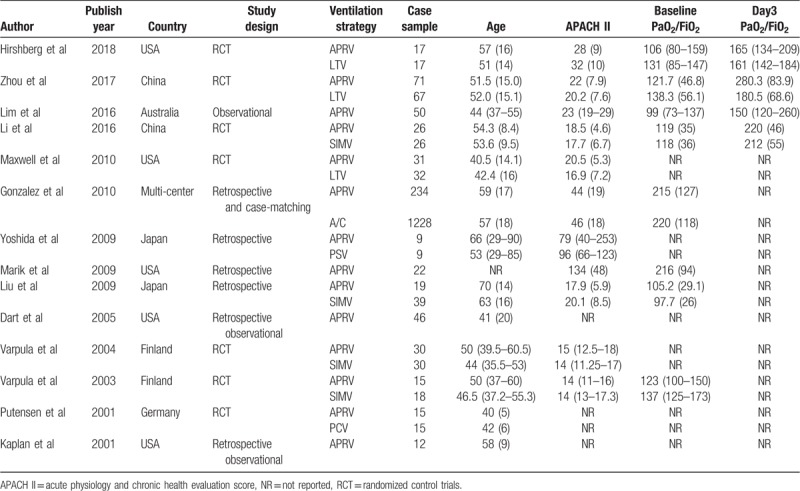
Characteristics of systematically reviewed studies.

### 3.3 Outcome assessment

**Figure 2:**
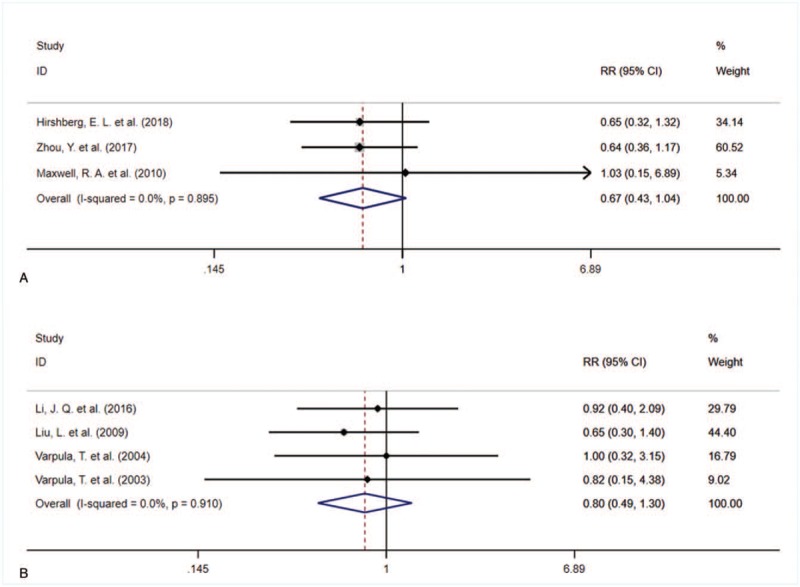
The forest plot comparing the mortality between APRV vs low tidal volume (A), and APRV vs synchronized intermittent mandatory ventilation (B). APRV = airway pressure release ventilation.

The outcomes and findings in included studies were summarized in Table 3, in which most studies suggested that the PaO_2_/FiO_2_ would increase significantly after 24 hours which could improve oxygenation and reduce the duration of ventilation and ICU stay. The forest plot comparing the mortality was shown in Figure 2. There were no significant difference in mortality between APRV vs LTV (RR = 0.67, 95%CI = 0.43–1.04, *P* = .073, Fig. 2A), and APRV vs SIMV (RR = 0.80, 95%CI = 0.49–1.30, *P* = .370, Fig. 2B).

**Table 3:**
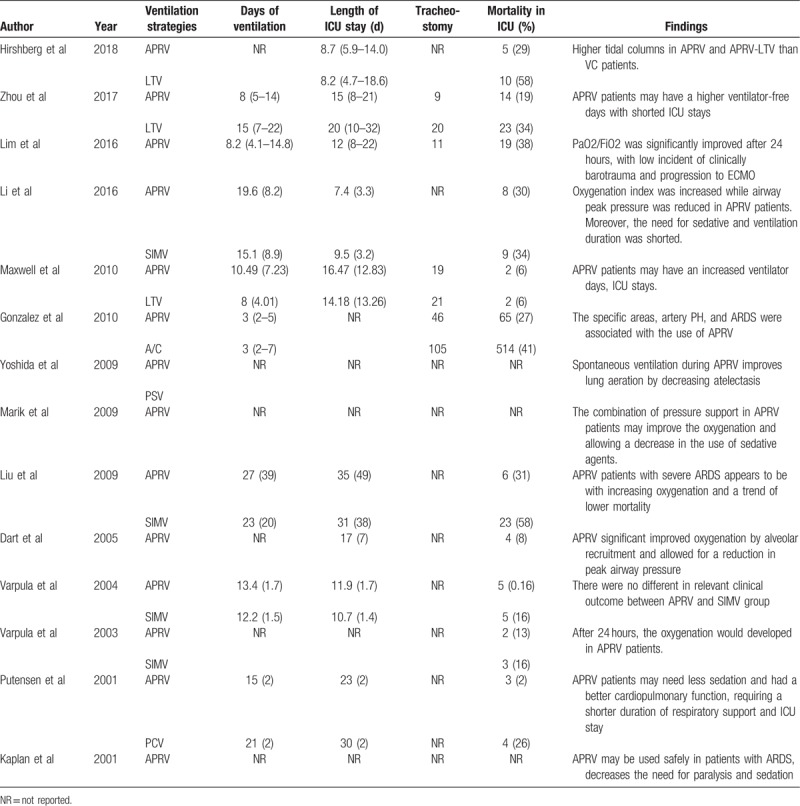
Outcome and findings in included studies.

**Figure 3:**
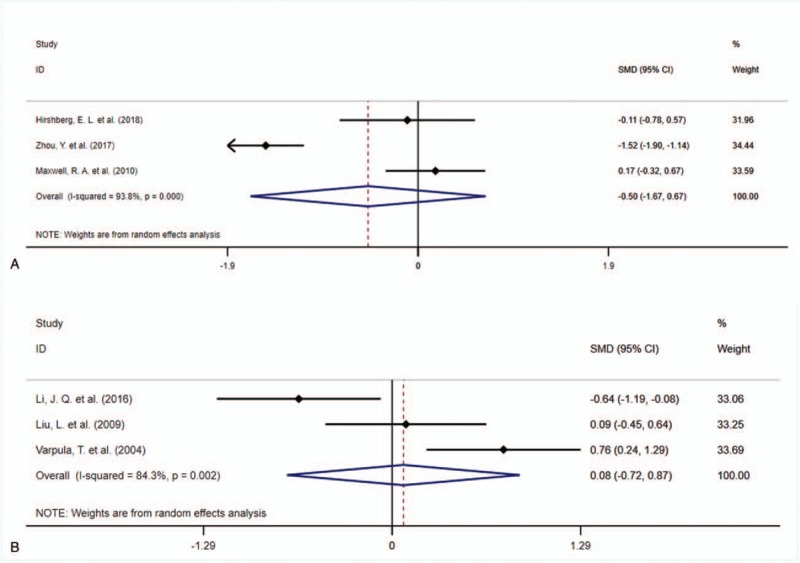
The forest plot comparing the duration of intensive care unit stay between APRV vs LTV (A), and APRV vs synchronized intermittent mandatory ventilation (B). APRV = airway pressure release ventilation.

The forest plot comparing the duration of ICU stay were plotted in Figure 3. Similarly, there were also no significant difference between APRV vs LTV (SMD = −0.50, 95%CI = −1.67–0.67, *P* = .404, Fig. 3A), and APRV vs SIMV (SMD = 0.08, 95%CI = −0.72–0.87, *P* = .849, Fig. 3B).

## 4 Discussion

This is the first systematic review and meta-analysis focusing on comparison the impact of APRV on patients with ARDS. Our study demonstrated that the APRV could increase the PaO_2_/FiO_2_ ratio significantly in ARDS patients. However, no evidence was demonstrated that APRV could decrease the mortality and duration of ICU comparing to SIMV and LTV.

The pathophysiology of ARDS results from acute inflammation affecting the alveolar-capillary membrane, which is related to intra-pulmonary and extra-pulmonary factors.^[29]^ Intra-pulmonary factors mainly include pneumonia and aspiration in patients; extra-pulmonary factors are severe infections caused by trauma or surgery, severe multiple injuries, and shock. Due to the acute onset of ARDS, patients usually have shortness of breath, accompanied by chest tightness, cough, and other symptoms. The patients with advanced disease may have altered consciousness and even a outcome of death.^[4]^ An increase in the permeability of the membrane was found with the recruitment of neutrophils and other inflammation factors, which resulted in the pulmonary edema.^[30]^ Some studies suggested that the pathogenesis of ARDS may also be associated with systemic inflammatory response syndrome (SIRS).^[31]^ When the patients suffer from infection, trauma, and stress, inflammatory factors, such as interleukin and tumor necrosis factor, are released, forming a “waterfall effect”, causing uncontrollable symptoms of inflammation in patients, and eventually leading to the occurrence of ARDS.^[32]^ Besides, the excessive activation of complement leads to an increase in alveolar vascular permeability and aggravation of exudation.^[33]^ Furthermore, ventilation/perfusion (V/Q) dysregulation is also an essential feature of ARDS.^[34]^ Because ARDS lesions tend to be heterogeneous, when the ventilator is used, alveolar ventilation is insufficient due to fibrosis. V/Q is down regulated which causing a physiological shunt. At the same time, due to severe hypoxemia, stasis of the microcirculation in the lungs, microthrombus formation, the hemodynamic changes leading to a relative upregulation of V/Q, which resulting in the increase of “dead space”.

In the early stage of MV application, several studies realized that the conventional MV could lead to the excessive expansion of the alveolar ventilation area, while the collapse area cannot be expanded as the ideal state. Moreover, the junction area is in an alternating state of expansion and collapse. As a result, conventional MV may not improve the patient's oxygenation, and sometimes even promote the progress of the disease. Afterward, the low tidal volume with high PEEP ventilation mode was introduced for ARDS.^[35]^ Since firstly proposed in 1987, the APRV experienced a flourishing development with pros and cons. APRV produces exhalation by releasing pressure from high pressure (P_high_) to low pressure (P_low_), with a higher baseline pressure favors oxygenation, and intermittent pressure release promotes CO_2_ excretion. By setting P_high_, APRV does not lead to an excessive alveolar pressure, but maintains the alveolar complex tension for a few seconds, avoiding excessive inspiratory lung volume resulting in excessive traction and physical damage to the alveoli; setting P_low_ in a short release phase, with a longer time to remain constant alveoli open, avoiding repeated opening and closing of the damaged alveoli.^[36]^ T_high_ is the time to use the CPAP to recapture the collapsed alveoli, while preventing lung damage caused by small airway periodic respiration to achieve optimal oxygenation and improve lung compliance. Usually, the T_high_ is set to 80% to 95% of the entire respiratory cycle, that is, the lowest value is 4.0 seconds. T_low_ allows both adequate ventilation and complete exhalation of constant alveolar, which results in PEEP facilitating alveolar recruitment. T_low_ is determined by the time constant of the expiratory flow rate rather than artificially set, which is more consistent with lung physiology and respiratory mechanics according to the expiratory flow rate and peak flow rate.^[37]^ The static pressure-volume curve can reflect the elastic characteristics of the lung. The low inflection point is considered to be the beginning of the collapse of alveolar in patients with ARDS. The high inflection point is the beginning of the collapse of alveolar recap, and the beginning of excessive alveolar expansion.^[38]^ Thereafter, APRV is also considered to be a “protective lung ventilation” strategy for preventing ventilator-induced lung injury (VILI). APRV is based on the “pulmonary open strategy”, which progressively retracts the collapsed alveoli by a higher airway pressure while preserving spontaneous breathing.^[38]^

A large number of animal experiments and clinical studies have shown that APRV can use a slightly higher airway pressure, a smaller peak inspiratory pressure, lower minute ventilation and fewer sedatives, to reduce dead space ventilation, increase alveolar ventilation, and improve oxygenation.^[36,39,40]^ Roy et al designed a rat model of trauma and found that the group using APRV had a significant decrease of PaO_2_/FiO_2_ in histopathology comparing to conventional MV group. Moreover, the bronchoalveolar lavage fluid total protein was decreased with a substantial increase of surfactant protein B concentration and epithelial cadherin tissue expression.^[39]^ In terms of VILI, Emr et al demonstrated that the APRV could prevent the VILI and ARDS comparing to the conventional MV and PEEP in healthy rats.^[40]^ Besides, APRV has little effect on the cardiovascular system, which could improve heart function to some extent, restore or approaching normal V/Q ratio, increase systemic blood flow, and improve the perfusion of the whole body and organ.^[41]^

In our study, the average increasing rate of PaO_2_/FiO_2_ was higher in APRV group which means after 3 days of APRV application, patients would get more oxygenation. Because of the limited number of clinical data, some results cannot be shown in the forest plot. However, in the findings of different studies, sedative agents are also decreasing with a reduction in peak airway pressure, and thereafter, it potentially reduces the time of ventilation and the duration of ICU stay. But unfortunately, no evidence could be demonstrated to support that APRV could decrease the mortality and the duration of ICU stay. However, APRV is still regarded as a relatively safe ventilation mode for patients with ARDS, and the application of appropriate parameters at specific disease stages can help improve outcomes. Even though there were several prospective clinical trials undergoing, the number of cases reported in the relevant research is currently small, which may be related to the lack of clear definition criteria.^[42,43]^ Therefore, it is urgent to establish a unified APRV parameter setting standard in the clinical settings, which is to maximize lung recruitment, improve oxygenation, and avoid VILI.^[44]^

There were still some limitations in our study. First, the whole sample of the ARDS patients using APRV was still scarce, and the meta-regression, meta-network cannot be undertaken in terms of lack of comparable studies. A large sample, multi-center, prospective randomized clinical trials are still needed. Secondly, due to the different definition of ARDS and ALI, and a different set of ARDS mode, some bias and heterogeneity cannot be avoided. The standardized setting mode of ARPV needs to be recommended. Thirdly, despite the data we included covering several countries, the studies we included were limited in English, and more European, African, and Asian studies should be searched in the local database.

## 5 Conclusion

In conclusion, the APRV protocol would have a higher increase in PaO_2_/FiO_2_ ratio, which may improve the oxygenation and thereafter potentially improve the symptoms of ARDS patients. APRV was a safe MV protocol with a compatible effect comparing to LTV and SIMV. Further investigation should be undertaken to investigate the standardized APRV setting and detect the ventilation mechanism in ARDS using the standardized definition.

## Author contributions

**Conceptualization:** Xuri Sun.

**Data curation:** Xuri Sun, Yuqi Liu, Neng Li.

**Formal analysis:** Neng Li.

**Methodology:** Neng Li, Deyuan You, Yanping Zhao.

**Software:** Yanping Zhao.

**Supervision:** Xuri Sun.

**Writing – original draft:** Xuri Sun.

**Writing – review & editing:** Yuqi Liu, Neng Li, Deyuan You, Yanping Zhao.
